# Electrophysiological characteristics and catheter ablation of ventricular arrhythmias arising from the superior septal left ventricle

**DOI:** 10.1186/s12872-024-03979-9

**Published:** 2024-06-24

**Authors:** Qifang Liu, Ye Tian, Zhi Jiang, Longhai Tian, Jing Huang, Ying Yang, Long Yang

**Affiliations:** Department of Cardiology, Guizhou Provincial People’s Hospital, 83 ZhongShan East Street, Guiyang, China

**Keywords:** Premature ventricular complex, Morphology, Spiky potential, Premature ventricular complex ablation

## Abstract

**Background and aims:**

Electrophysiological characteristics and radiofrequency catheter ablation (RFCA) of premature ventricular contractions (PVCs) originating from the superior septal left ventricle (SSLV) have not yet been fully characterized.

**Methods and results:**

This study included 247 patients who underwent RFCA for PVCs arising from the ventricular outflow tract between February 2020 and August 2022. The successful ablation site was on the SSLV in 37 of the 247 patients. In 12 (32.4%) of those 37 patients, a low amplitude and high frequency spiky potential (SP) was recognized. Five patients showed a narrow QRS duration (86.8 ± 4.6 ms), with a discrete SP observed in PVCs and sinus rhythm, which showed an isoelectric line with the ventricular electrogram at the earliest activation site. Seven patients showed a wide QRS duration (131.6 ± 4.5 ms), with SP observed in PVCs without an isoelectric line with the ventricular electrogram. RFCA was successful at the site of the earliest SP in all 12 patients. The time from SP onset at the successful ablation site to the QRS onset (local activation time) was 30 ± 12 ms, which differed significantly from that for the remaining 25 patients withoutSP(22.1 ± 7.1 ms, *P* < 0.05).

**Conclusions:**

SPs were recorded in 12 (32.4%) of the 37 patients with PVCs originating from the SSLV. The morphology of the PVCs may show a narrow or wide QRS duration and the target site for successful ablation should be identified by the earliest SP.

**Supplementary Information:**

The online version contains supplementary material available at 10.1186/s12872-024-03979-9.

## Introduction

Premature ventricular complexes (PVCs) are one of the most encountered cardiac arrhythmia. PVCs with a burden of > 20% may be independently associated with PVC-induced cardiomyopathy [1]. Catheter ablation is more efficacious than medicines to treat PVCs in patients presenting with monomorphic premature complexes. Success of PVC ablation procedures range from approximately 80–95% [2].

**Fig.1:**
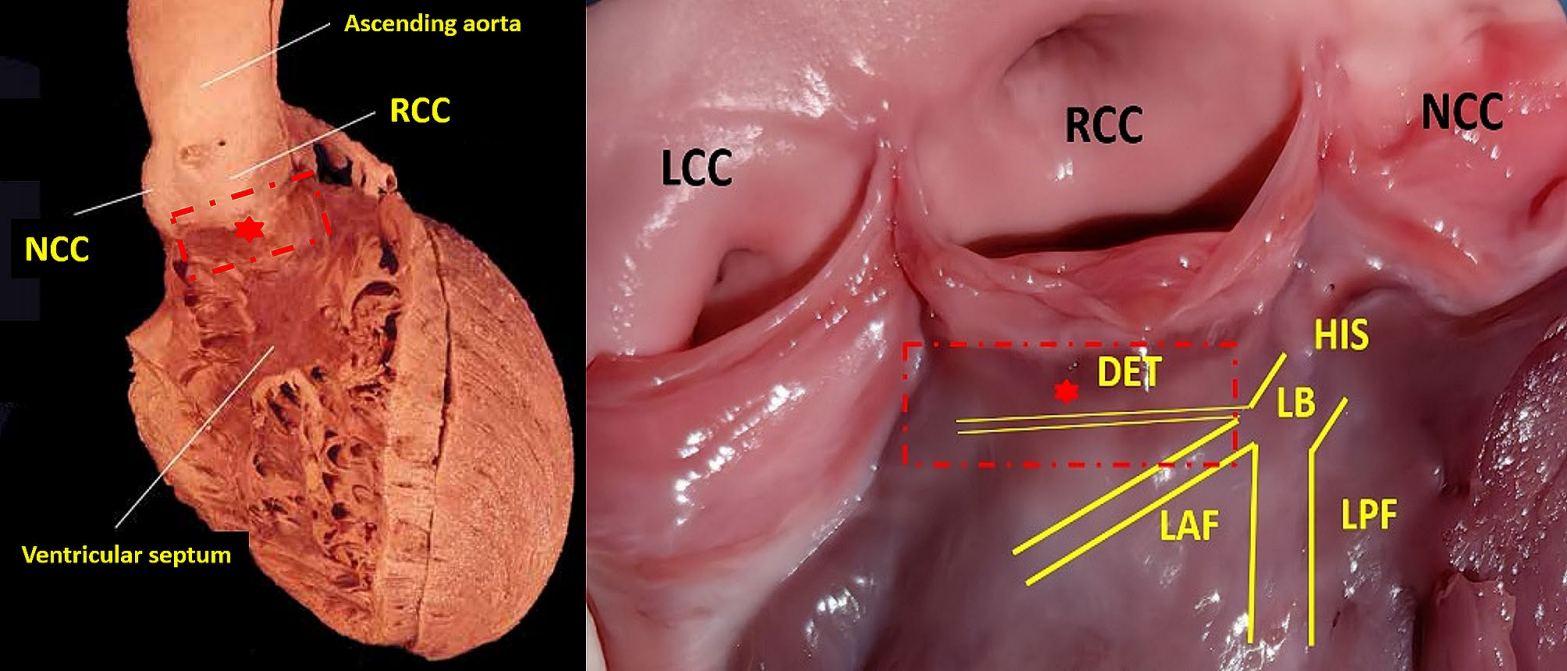
The anatomical structure of SSLV. Left panel The SSLV (red dashed box)viewed from the right ventricular and the anterior direction, which supports the right coronary aortic sinus. Right panel. The SSLV viewed from the endocardium of left ventricular, which includes DET, LAF and ventricular myocardium. DET: dead-end tract; RCC: right coronary cusp; LCC: left coronary cusp; NCC: noncoronary cusp; LB: left bundle; LAF: left anterior fascicular; LPF: left posterior fascicular; SSLV: superior septal left ventricular

The superior muscular ventricular septum supports the right coronary aortic sinus. The PVC originating from the superior septal left ventricle (SSLV), which is defined as the site of earliest electrical activation, corresponds to the right coronary cusp (RCC) and the right–left sub-valvular interleaflet triangle (Fig. 1). The mechanism behind PVCs originating from the SSLV remains elusive, and it could be influenced by ventricular myocardium or His-Purkinje fibers lying within the SSLV [3–5]. In this study, we mainly explored the electrophysiological characteristics and ablation outcomes of premature ventricular contractions (PVCs) originating from the SSLV, based on current knowledge.

## Methods

### Study population

**Fig. 2:**
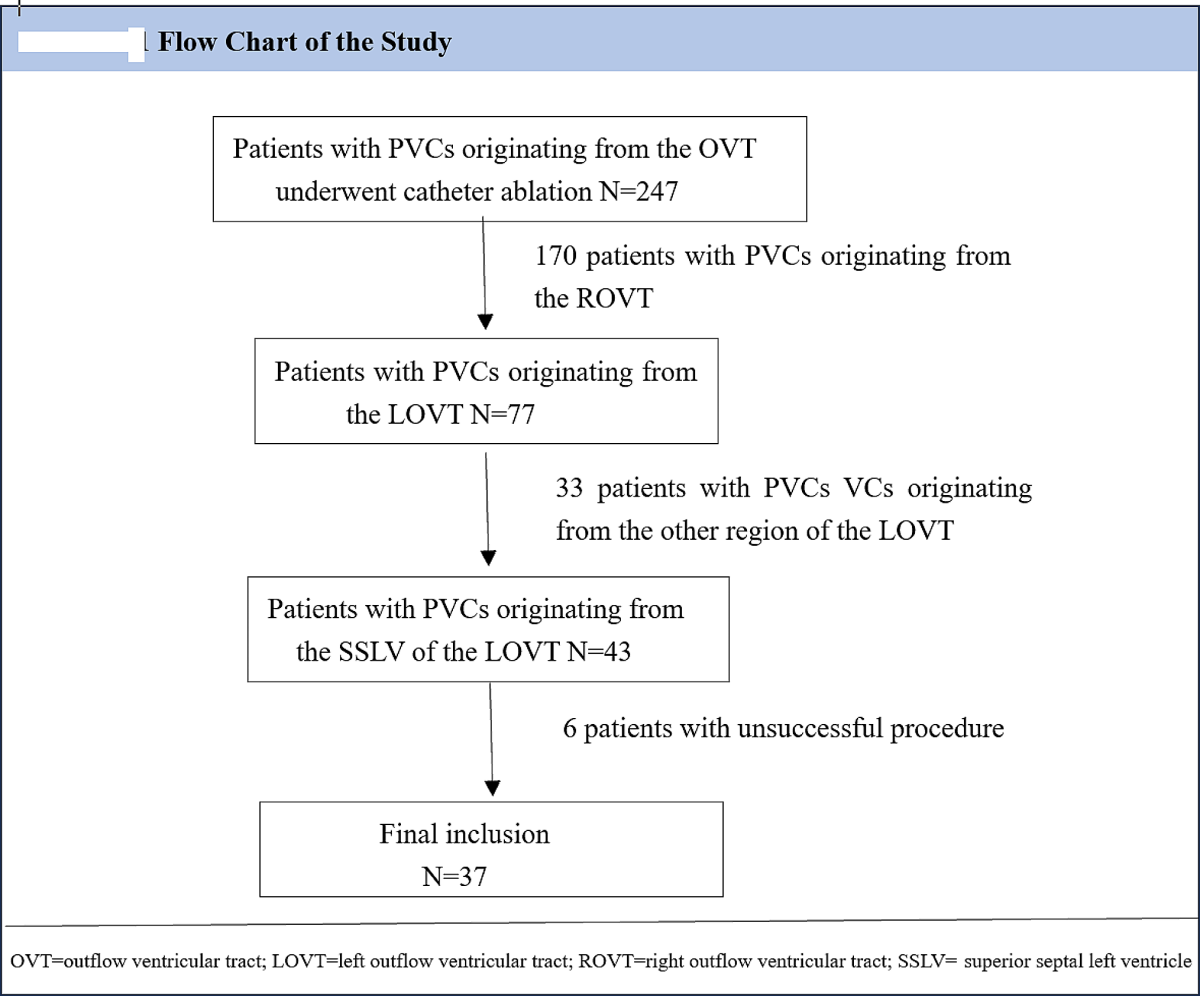
Flow chart of the study

Between February 2020 and August 2022, 247 patients with PVCs originating from the ventricular outflow tract underwent catheter ablation at our hospital. All patients provided written informed consent before the procedure, and the study was approved by the Institutional Review Board of Guizhou Provincial People’s Hospital. All methods were performed in accordance with the 2017 American Heart Association/American College of Cardiology/Heart Rhythm Society (AHA/ACC/HRS) guidelines for management of patients with ventricular arrhythmia. A comprehensive clinical evaluation, including 12-lead electrocardiography (ECG) and transthoracic echocardiography, was performed in all patients. Structural abnormalities were excluded using coronary angiography and cardiac magnetic resonance imaging when needed. The findings for 37 patients with successful ablation sites at the SSLV were retrospectively analyzed using electrophysiological mapping and radiofrequency catheter ablation (RFCA) (Fig. 2). The ECG characteristics of PVC exhibited a wide QRS morphology in 32 patients and a narrow QRS morphology in 5 patients. For PVC showing narrow QRS pattern, the QRS morphology was characterized by qR in leads II, II, and aVF, an rS or RS pattern in leads V1, a QS pattern in leads aVR and aVL, and a precordial transition in lead V2 and V3. For PVC showing wide QRS pattern, the QRS morphology was characterized by R in leads II, II, and aVF, an rS, Rs and R pattern in leads V1, a QS pattern in leads aVR and aVL, and a precordial transition in lead V1 and V2. Patients in whom ablation failed and PVC was eliminated by ablation from adjacent anatomic structures, including the left coronary cusp, the septal RVOT and great cardiac veins were excluded. Data collection and analysis were approved by the Institutional Review Board of Guizhou Provincial People’s Hospital. The electrophysiological studies and clinical outcomes are reported below.

### Electrophysiological studies and catheter ablation

Mapping and ablation were attempted at the SSLV, via a femoral artery approach. Heparin was administered to maintain activated clotting times of 250–350 s, while the catheters were positioned in the systemic circulation. Activation mapping was performed using a 3.5-mm irrigated-tip quadripolar ablation catheter (ThermoCool; Biosense Webster). Electroanatomical mapping was performed using CARTO (Biosense Webster). BiEGMs (filtered at 30–400 Hz) were recorded from the distal (M1–M2) electrode pair of the ablation catheter, while UniEGMs (filtered at 1–240 Hz, Wilson’s central terminal) were recorded from the distal electrodes M1 and M2. In the presence of a spontaneous clinical PVC, activation mapping was performed using the surface PVC QRS as the temporal reference. The mapping strategy focused on the earliest low amplitude and high frequency spiky potentials(SPs). The conduction interval from the earliest SPs to the onset of the QRS was defined as the earliest activation time in patients with SPs. Local EGM to QRS onset for both bipolar and unipolar was defined as the activation time in patients without SPs. If no spontaneous PVC occurred, intravenous isoproterenol (2–8 µg/min) infusion was administered. If the PVC was non-inducible, the case was excluded.

In all patients, the location of the coronary artery ostia was identified using angiography before ablation to avoid coronary artery complications. Ablation was performed using an irrigated catheter with a power output of 30 W and a maximum power setting of 50 W. When the incidence of PVCs reduced during the first 10 s of application, RF delivery was continued for 90 s while carefully monitoring the conduction intervals. The procedural endpoint was reached when clinical PVCs did not occur spontaneously after a waiting period of 30 min with intermittent isoproterenol infusion.

### Statistical analysis

Statistical analyses were performed using GraphPad Prism 9.4. Statistical significance was set at *P* < 0.05. Comparisons for continuous variables were performed by Student’s t-test for variables that follow a normal distribution. Continuous variables are expressed as mean ± standard deviation (SD). Categorical variables are expressed as numbers and percentages and analyzed using the chi-square test.

### Follow-up assessments

All patients underwent 24-hour Holter monitoring on the day after the procedure and were followed up in the cardiology outpatient clinic. ECG and 24-h Holter monitoring were performed every three months for the first year and every six months thereafter. The patients were not administered antiarrhythmic drugs after RFCA.

## Results

### Baseline and procedural characteristics

**Fig. 3:**
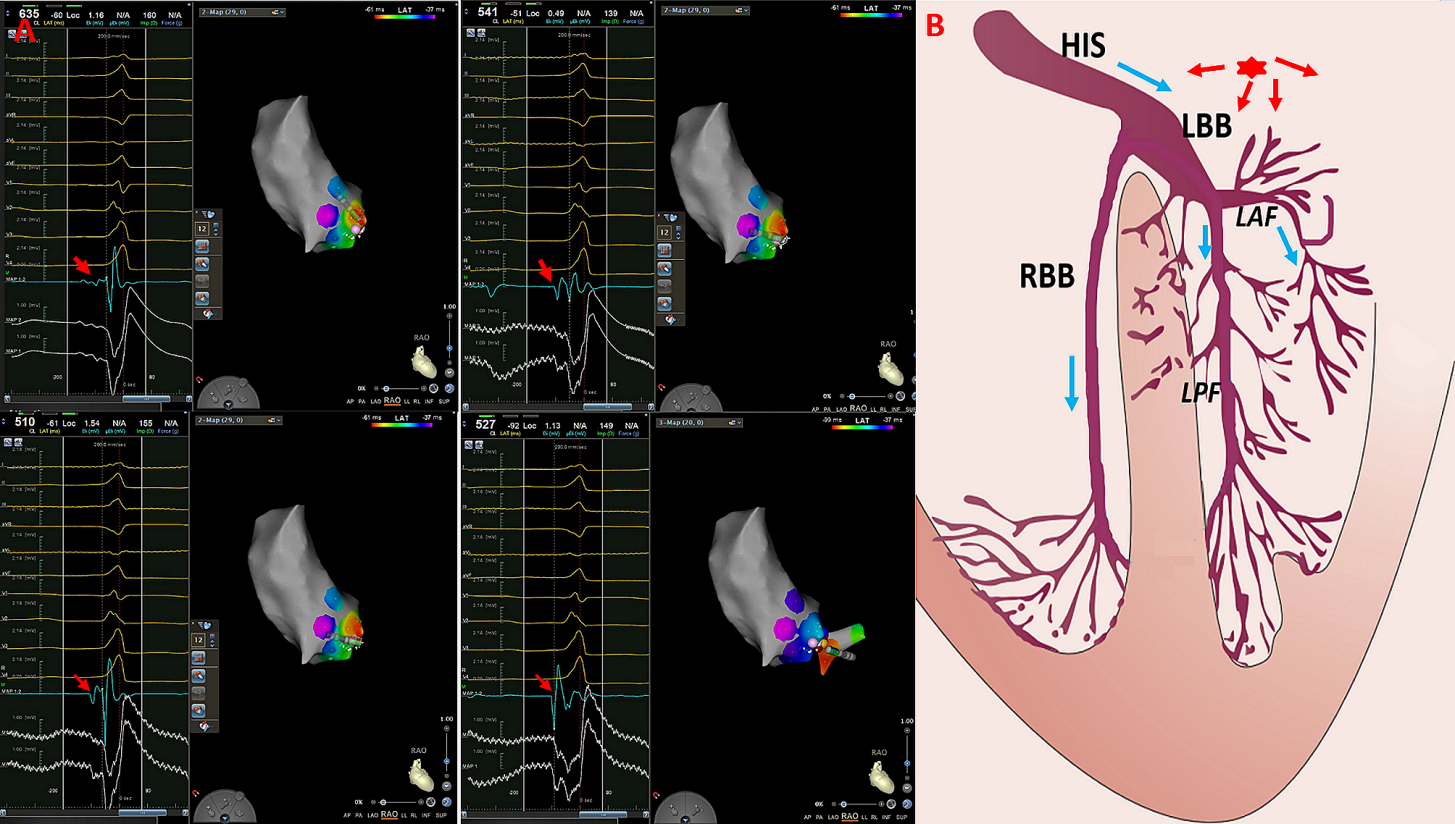
The Successful Ablation for PVC originating from ventricular myocardium and showing wide QRS complex. Panel A Electro-anatomical mapping showing the earliest ventricular activation site of PVC at the right–left sub-valvular interleaflet triangle. The bipolar electrogram amplitude changes substantially from low to high (far-feild potential to near-field potential) when moving the catheter from remote zones to the origin site of PVC. Panel B Model diagrams showing the origin site(red star) of PVC originating from ventricular myocardium of SSLV and ventricular activation sequence during PVCs (red arrow) and sinus rhythm (blue arrow). RCC: right coronary cusp; SSLV: superior septal left ventricular

A total of 247 patients with PVCs between February 2020 and August 2022 were included. The successful ablation site was at the SSLV in 37 patients (14.9%). All 37 patients had symptomatic PVCs and were refractory to therapy with 1–2 antiarrhythmic drugs. The incidence of a PVC burden on 24-h Holter monitoring was 24.8% ± 11.3%, while that of normal LV ejection fraction on echocardiography was 56.8% ± 7.3%. In 12 of the 37 patients, the low amplitude and high frequency SPs could be recorded at the successful ablation site. In the remaining 25 patients, SPs were not observed at the successful ablation site (Fig. 3). No significant differences were observed in age, PVC burden, and LVEF between patients with and without SPs. The baseline clinical characteristics of the patients are presented in Table 1.

**Table 1:** Comparison of Characteristics Between Patients with and without spiky potentials (SPs)

	age	sex	PVC burden	LVEF	Antiarrhythmic	LAT	Number
	(years)	(Male)	mean±SD	(%)	drugs (type)	(ms)	RF application
SPs+ (*N*=12)	37.8±16.4	66.7%	26.3±12.4	58.3±6.4	1.2±0.5	30.1±8.3	1.7 ± 0.8
SPs- (*N*=25)	40.1±19.2	56.0%	23.8±13.9	55.8±7.6	1.5±0.7	22.1±7.1	2.8 ± 1.6
P value	*P*=0.48	*P*=0.89	*P*=0.35	*P*=0.18	*P*=0.24	*P*<0.05	*P*<0.05

SPs+ indicates Patients with SPs; SPs- indicates Patients without SPs; LVEF: left ventricular ejection fraction; PVC: premature ventricular complex; LAT: local activation time; Anti-arrhythmic drugs include β-blockers, CCBs, mexiletine, amiodarone and propafenone

Among the 12 patients with low amplitude and high frequency SPs, five presented narrow QRS morphology and the mean QRS duration of the PVCs was 86.8 ± 4.6 ms, which differed significantly from that in patients without SPs (138.9 ± 14.2 ms, *P* < 0.001). The remaining seven patients presented a wide QRS morphology and the mean QRS duration of the PVCs in these patients was 131.6 ± 4.5 ms. which was not significantly different from that in patients without SPs (*P*>0.05; Table 2).

**Table 2:** Electrophysiologic study characteristics in patients

	QRS duration (ms)	SP-V interval in PVC (ms)	SP-V interval in SR (ms)
SPs- (*N*=25)	138.9±14.2	**−**	**−**
SPs+ with narrow QRS (*N*=5)	86.8±4.6	38.0±4.9	28.4±2.3
SPs+ with wide QRS (*N*=7)	131.6±4.5	24.4±4.4	

*P*< 0.01 SPs- vs. SPs+ with narrow QRS for QRS duration

*P*=0.192 SPs- vs. SPs+ with wide QRS for QRS duration

SPs+ indicates Patients with SPs; SPs- indicates Patients without SPs; SP-V interval: time from SPs to ventricular electrogram onset; SR: Sinus rhythm

### Electrophysiologic studies and RFCA

Of the 37 patients whose successful ablation site was at the SSLV, SPs were recorded in 12 patients (32.4%), 5 (5/37, 13.5%) at the RCC and 7 underneath the RCC (7/37, 18.9%). SPs were not observed at the successful ablation site in the remaining 25 patients: 11 (11/37, 29.7%) at the RCC and 14 underneath the RCC (14/37, 37.8%). The activation time (time from onset of the SPs to the QRS onset, SP-V interval) recorded at the successful ablation site differed significantly between patients with SPs (30.1 ± 8.3 ms) and those without SPs (22.1 ± 7.1 ms; *P* < 0.05). The total number of RF ablation applications was 1.7 ± 0.8 for patients with SPs and 2.8 ± 1.6 for patients without SPs (*P* < 0.05); accordingly, the mapping points per ventricular arrhythmia were 28 ± 13 and 45 ± 20 (*P* < 0.05), respectively (Table 1). Additional mapping was required at adjacent anatomic sites, including the LCC, septal RVOT, and GCV, in 14 of 25 patients without SPs and in one of 12 patients with SPs(56.0% vs. 8.33%, *P* < 0.001).

**Fig. 4:**
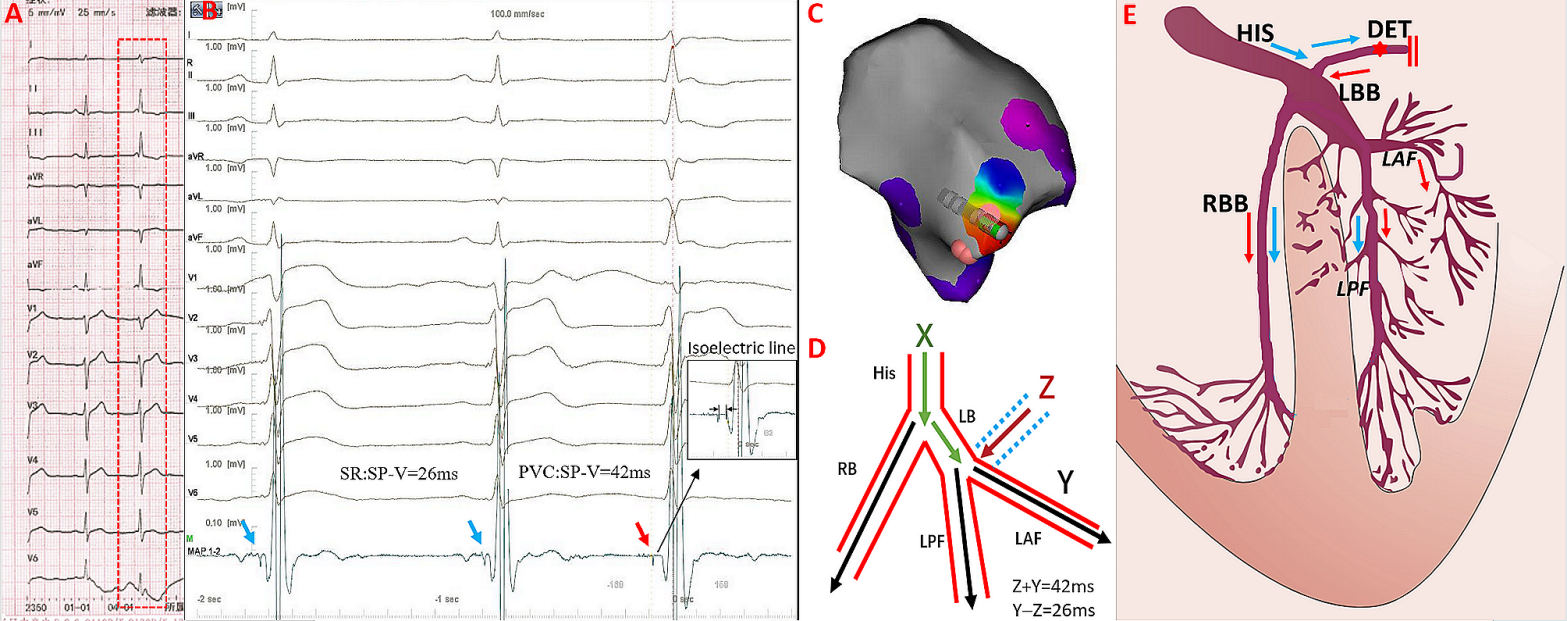
The Successful Ablation for PVC showing narrow QRS complex with SP and isoelectric line. Panel A The baseline 12-lead ECG showed PVC with the QRS duration 97ms. Panel B and C Electro-anatomical mapping showing the earliest ventricular activation site of PVC at the RCC. Target electrogram shows SPs (blue arrow and red arrow) with SP-V interval of 26 ms in sinus rhythm and SP-V interval of 42 ms in PVCs and the isoelectric line between SP and ventricular potential. Panel D Model diagram showing the mechanism of SP-V interval in sinus rhythm is shorter than that of PVCs at the earliest activation site. Panel E. Model diagrams showing the origin site of PVC (red star) and ventricular activation sequence during PVCs (red arrow) and sinus rhythm (blue arrow). SP: spiky potential; RCC: right coronary cusp

**Fig. 5:**
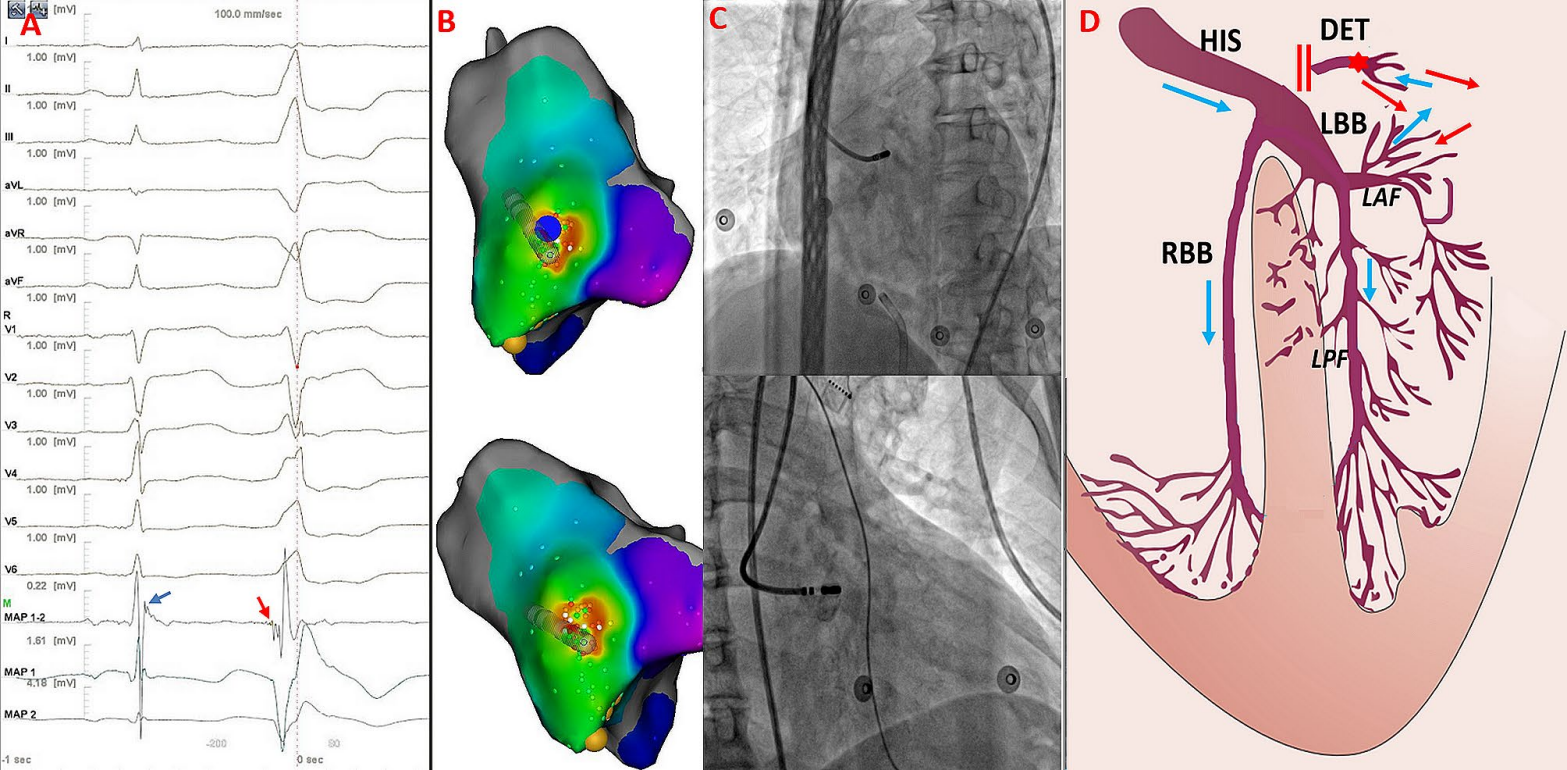
The Successful Ablation for PVC showing wide QRS complex(127ms)with SP and no isoelectric line. Panel A and B Electro-anatomical mapping showing the earliest ventricular activation site of PVC at RCC. Target electrogram shows the earliest SP (red arrow) with SP-V interval of 28 ms in PVCs and SP (blue arrow) following the local ventricular potential during sinus rhythm, suggesting a shift in timing. Panel C RAO and LAO radiographic views showing ABL at the RCC. Panel D Model diagrams showing the origin site of PVC (red star) and ventricular activation sequence during PVCs (red arrow) and sinus rhythm (blue arrow). SP: spiky potential; RCC: right coronary cusp; RAO: right anterior oblique; LAO: left anterior oblique

Among the 12 patients with SPs, SPs preceding the QRS were observed both in sinus rhythm and PVCs in five patients with a narrow QRS duration (Fig. 4). An isoelectric line was observed between the end of the SP and onset of the ventricular electrogram, and the SP-V interval was greater in PVCs than in sinus rhythm (38.0 ± 4.9 ms vs. 28.4 ± 2.3 ms, *P* < 0.01). RF energy application resulted in SPs automaticity with similar SP-V interval and identical QRS morphology to the PVC (supplementary file). In the remaining seven patients, the PVC morphology exhibited a wide QRS duration. SPs were recorded at the terminal portion of the ventricular potentials during sinus rhythm, which showed an inversion of the sequence during the PVC in three patients. These potentials were immediately followed by the ventricular potential during PVCs, leaving no room for an isoelectric line (Fig. 5). SPs could be recorded in PVCs, but not in sinus rhythm at the earliest activation site in the other four patients. The SP-V interval recorded at the successful ablation site differed significantly between patients with narrow and wide QRS durations (38.0 ± 4.9 ms vs. 22.1 ± 7.1 ms, *P* < 0.05; Table 2). The electrophysiological characteristics of the 12 patients are shown in Table 3.

**Table 3:** Electrophysiologic study characteristics in patients with SPs

CASE during PVC	QRS duration during SR	SP-V interval of potential	SP-V interval	Inversion	Isoelectric line
1	91ms	42ms	26ms	**−**	+
2	83ms	35ms	30ms	**−**	+
3	91ms	37ms	29ms	**−**	+
4	88ms	32ms	26ms	**−**	+
5	81ms	44ms	31ms	**−**	+
6	140ms	21ms	−	**−**	+
7	130ms	19ms	−	**−**	**−**
8	131ms	28ms	Following V	+	−
9	128ms	30ms	Following V	+	−
10	127ms	28ms	−	−	−
11	130ms	29ms	Following V	+	−
12	135ms	34ms	−	−	+

SP-V interval: time from SPs to ventricular electrogram onset; − indicates loss of inversion of potential and isoelectric line

## Discussion

Several studies have explored the electrophysiological characteristics of PVCs with successful ablation sites in the RCC and the right–left sub-valvular interleaflet triangle [6–8]. However, the electrophysiological characteristics and catheter ablation of PVCs originating from SSLV have not yet been fully characterized. There were three major findings for SSLV-PVCs in this study. First, SPs were recorded not only in PVCs with narrow QRS duration, but also in PVCs with wide QRS duration. Second, PVCs showed narrow QRS duration in five (13.5%) of 37 patients and the SP-V interval during PVCs was significant longer than that during sinus rhythm. Third, the mapping points and number of RFCA applications needed to terminate the PVCs were increased in PVC without SPs at the earliest activation site.

In this study, SPs were observed in five patients with PVCs showing a narrow QRS complex and in seven patients with PVCs showing a wide QRS complex. SPs were not observed in 25 patients with PVCs showing a wide QRS complex. Chen et al. reported that sharp, high-frequency and presystolic potentials were observed in PVCs originating from proximal left anterior fascicle (LAF), which presented narrow QRS duration and could be successfully ablated from the RCC [6]. Hachiya et al. reported that an isolated spiky prepotential could be observed at the successful ablation site for PVCs with a wide QRS duration, which could be successfully ablated from the RCC and a dead-end tract (DET) was a plausible explanation for these discrete prepotentials [5]. In our study, SPs could be recorded on successful ablation sites for PVCs originating from the SSLV. Although we did not confirm whether these low amplitude and high-frequency potentials represent the activation of the myocardium or His-Purkinje system in the SSLV, it is important to understand if SPs would be a sufficient indicator for successful ablation of PVCs with different QRS durations [9].

In our patients with PVCs showing a narrow QRS complex, SPs occurring slightly before the ventricular electrogram with an isoelectric line were recorded at the SSLV. The route of the DET and the proximal LAF lies within the anatomical structure of the SSLV [10–12]. Furthermore, the characteristics of these recorded potentials seem to be qualitatively similar to those of the His-Purkinje origin. Therefore, the LAF or DET might have been sites of origin for the ectopic arrhythmia foci in our study. Previous studies have reported that SPs in the SSLV represent the proximal LAF and that the SP-V interval in sinus rhythm is equal to the SP-V interval of PVCs at the earliest activation site for PVCs originating from LAF [6, 13]. Jinglin Z et al. reported that SPs in the SSLV represent the activation of DET, with the SP-V interval in sinus rhythm showing no difference from the SP-V interval of PVCs at the earliest activation site [3]. In our study, the SP-V interval in sinus rhythm was significantly shorter than that of PVCs at the earliest activation site. We deduced that the ectopic focus was only electrically connected the distal LBB or the proximal LAF, having no electrical connection with the ventricular myocardium. Premature contraction activates the His-Purkinje system and ventricular myocardium by reverse conduction to the distal LBB or proximal LAF, resulting in a shorter SP-V interval in sinus rhythm at the earliest activation site than that of the PVCs (Fig. 3). Therefore, DET, instead of the proximal LAF, seemed to be the true origin of these PVCs.

In the patients without SPs recorded in the earliest activation site for PVCs mapping, the PVC morphologies presented a wide QRS duration. More mapping areas were needed than those for the patients with SPs recorded in the SSLV to identify an arrhythmogenic focal source. Accordingly, the adjacent anatomic area of mapping included the LCC, RVOT, and GCV in many patients without SPs recorded [14, 15]. Furthermore, SPs were observed in seven patients with PVCs showing a wide QRS complex in our study. In contrast to the PVCs without SPs recorded in the earliest activation site, it is easy to achieve the successful elimination of PVCs. We speculated that the Purkinje fibers might be the real source of the ectopic activity, which is not electrically connected the distal LBB or the proximal LAF but rather to the ventricular myocardium of the SSLV. In this situation, PVCs presented wide QRS durations and were easily eliminated with RF applications. Certainly, further basic research is needed for better understanding of the real origin of such SPs. Furthermore, high density mapping catheter is very important to collect a greater amount of data for SPs. However, high density mapping catheter manipulation is more easily than catheter ablation in resulting in mechanically induced PVCs and mechanical suppression of clinical PVCs. In the near future, it is hoped that high density mapping will enter clinical prime time for PVCs mapping and ablation.

### Study limitations

First, the SP, seemingly originating from the conduction system, was only recorded at the SSLV. However, there is no direct evidence indicating that the SPs represent DET, the proximal LAF, or myocardium. Additional studies are necessary to elucidate this mechanism. Second, this study did not include unsuccessful cases. Regarding the unsuccessful cases, we found that no SP was observed in the SSLV. Third, it was the small number of population and a single center non-randomized analysis. Further population-based data are required to clarify the actual prevalence of SPs in the SSLV.

## Conclusion

A SP might be detected at earliest activation site in PVCs originating from the SSLV. The morphology of such PVCs may show narrow or wide QRS durations and the target site for successful ablation should be identified by the earliest SP. Understanding such electrophysiological characteristics has been suggested to allow reducing the anatomic area of mapping and the number of RFCA applications.

### Electronic supplementary material

Below is the link to the electronic supplementary material.

Supplementary Material 1

## Data Availability

The data that support the findings of this study are available in this published article and its supplementary information files.
