# Supplementary material for: Electrophysiological characteristics and catheter ablation of ventricular arrhythmias arising from the superior septal left ventricle
Source: BMC Cardiovasc Disord. 2024 Jun 24;24:316. doi: 10.1186/s12872-024-03979-9 (PMC11194864; doi:10.1186/s12872-024-03979-9)
Supplement: Supplementary file 1 — Supplementary Material 1 [file 12872_2024_3979_MOESM1_ESM.docx]

Supplementary file


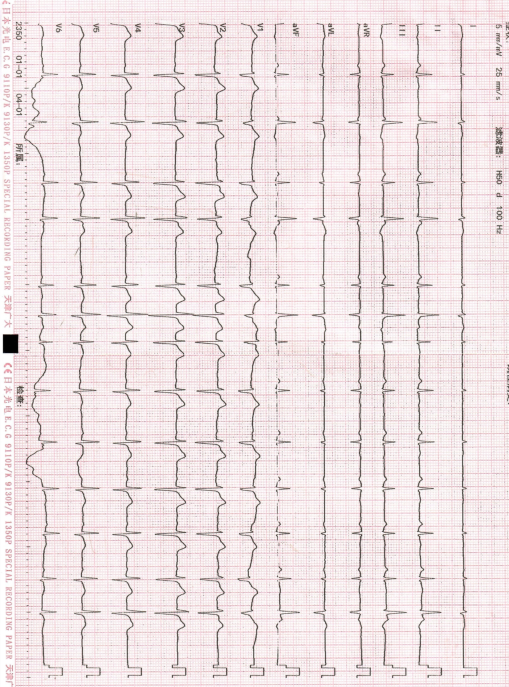


ECG shows a sinus rhythm with frequent PVCs with QRS duration 97ms（red arrow）


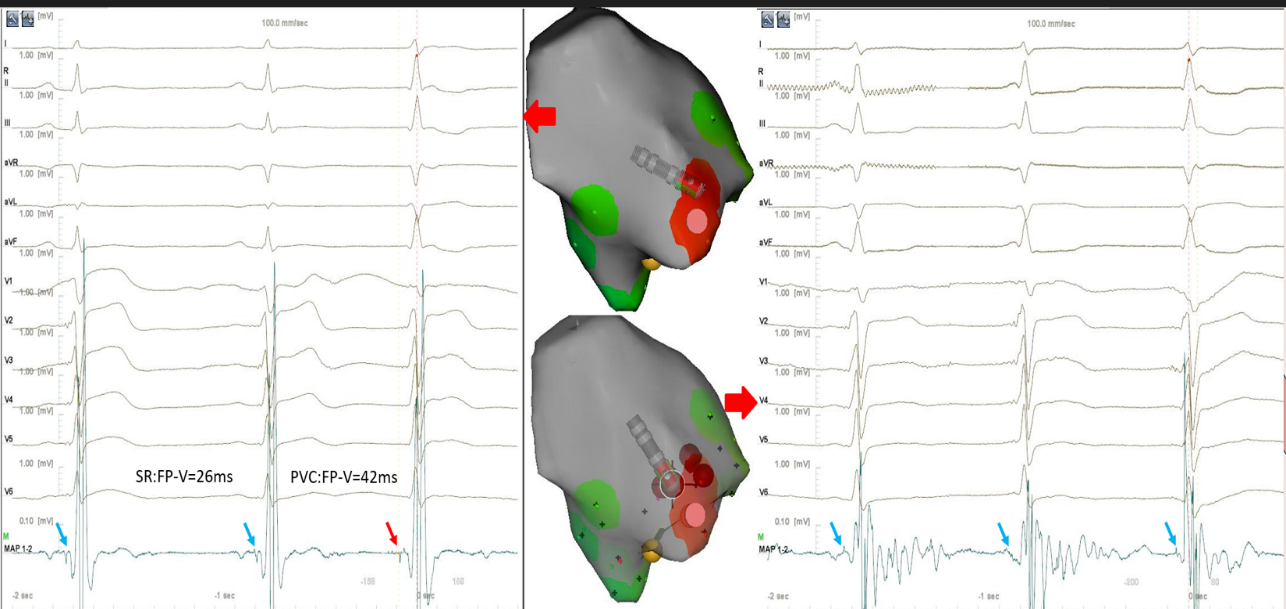


Left panel: Target electrogram shows spiky potentials (SPs) (blue arrow) with SP-V interval of 26 ms in sinus rhythm and the earliest SP (red arrow) with SP-V interval of 42 ms in PVCs. Right panel: RF energy application inducing SP automaticity (blue arrow) with identical QRS morphology to the PVC
